# Emergence and circulation of enterovirus B species in infants in southern China: A multicenter retrospective analysis

**DOI:** 10.1080/21505594.2024.2329569

**Published:** 2024-03-31

**Authors:** Xiaohan Yang, Yudan Wu, Hongyu Zhao, Pan Liu, Lihua Liang, Aihua Yin

**Affiliations:** a Medical Genetic Center, Guangdong Women and Children Hospital, Guangzhou 511400, China; b Department of Clinical Laboratory, the First Affiliated Hospital of Henan University of Science and Technology, Luoyang 471003, China

**Keywords:** Enterovirus, epidemiology, infants, perinatal mother-to-child transmission, southern China

## Abstract

**Background:**

Enteroviruses (EV) are common and can cause severe diseases, particularly in young children. However, the information of EV infection in infants in China is limited due to the vast population size and extensive geographical area of the country. Here, we conducted a retrospective multicenter analysis of available EV data to assess the current epidemiological situation in the infant population in southern China.

**Methods:**

The study enrolled infants with suspected EV infection from 34 hospitals across 12 cities in southern China between 2019 to 2022, and the confirmation of EV was done using RT-PCR and VP1 gene sequencing.

**Results:**

Out of 1221 infants enrolled, 330 (27.03%) were confirmed as EV-infected. Of these, 260 (78.79%) were newborns aged 0–28 days. The EV belonged to three species: EV-B (80.61%), EV-A (11.82%), and human rhinovirus (7.58%). Newborns were more susceptible to EV-B than older infants (*p* < 0.001). Within EV-B, we identified 15 types, with coxsackievirus (CV) B3 (20.91%), echovirus (E) 11 (19.70%), and E18 (16.97%) being the most common. The predominant EV types changed across different years. EV infection in infants followed a seasonal pattern, with a higher incidence from May to August. Furthermore, perinatal mother-to-child EV transmission in 12 mother-newborn pairs were observed.

**Conclusion:**

Our study is the first to demonstrate the emergence and widespread circulation of EV-B species, mainly CVB3, E11, and E18, in southern China, primarily affecting young infants. This research provides valuable insights for future epidemic assessment, prediction, as well as the elimination of mother-to-child transmission.

## Introduction

Enteroviruses (EV) are single-stranded positive-sense RNA virus belong to family *Picornaviridae*. According to the genetic divergence, EV infecting humans are assigned to four EV species (A – D) and three human rhinovirus (HRV) species [1]. EV-A, such as coxsackievirus (CV) A4, CVA5, CVA6, CVA10, CVA16, and EV-A71, primarily affects children and result in hand-foot-mouth disease (HFMD) and other neurological and systemic symptoms. EV-B mainly consists of echovirus (E), CVB, and CVA9 types, and often cause more severe and potentially fatal symptoms, including sepsis-like disease, meningitis, hepatitis with coagulopathy, and severe pneumonia [2]. Additionally, EV-B have the potential to cause hospital infection outbreaks among newborns, leading to high mortality rates [3].

EV have a global distribution, and a series of outbreaks across the Asia-Pacific region have been reported [4,5]. The incidence and circulation of EV types vary significantly by region and year, especially in infants. Surveillance in the United States showed that EV-B was the most common species isolated from infants and that the types changed over time [6,7]. In Europe, E30, E9, and E6 caused large epidemics every 5–6 years in young children with sepsis and meningitis [8–10]. In Asia, EV-B-associated outbreaks with high morbidity and mortality occurred frequently in recent years [3,11–13].

In response to the EV-A71 outbreaks, China established the national enhanced surveillance system (NESS) for HFMD in 2008. However, HFMD primarily manifests in children younger than 5 years with symptoms such as fever and rashes on the palms, soles, and oral mucosa. Infants, on the other hand, rarely exhibit rashes [14]. As a result, the NESS often fails to detect EV infection in infants, and there is limited information available on the prevalent types of EV in this population. Here, we aimed to address this gap by collecting and analysing data on EV from suspected paediatric patients in southern China over a 4-year period. The objective was to evaluate the epidemiological characteristics of EV infection in infants in this region.

## Materials and methods

### Study subjects

A retrospective multicenter analysis was conducted at 34 hospitals in 12 cities across southern China, from 1 April 2019 to 31 December 2022. These hospitals were located in Guangxi Zhuang Autonomous Region, Hunan Province, and Guangdong Province (Supplementary Figure S1). Infants who presented with sepsis-like disease, meningitis, myocarditis, HFMD, or other symptoms compatible with EV infection were enrolled in the study. Biological samples (stool, cerebrospinal fluid [CSF], and blood) from these infants were collected and sent to the Virology Department of Guangdong Women and Children Hospital for EV detection. Verbal informed consent was obtained from the parents or legal guardians of each child before enrolment. The study was approved by the ethics committee of Guangdong Women and Children Hospital (No. 202301319).

### EV detection and type determination

Nucleic acid extraction from samples was performed with an automated nucleic acid extraction system (Smart32, DAAN Gene Co., Ltd., Guangzhou, China) using the MagPure Universal RNA Precast Kit (Magen Biotechnology Co., Ltd., Guangzhou, China). EV RNA was detected by RT-PCR method using the Detection Kit for Enterovirus RNA (PCR-Fluorescence Probing) (DAAN Gene Co., Ltd., Guangzhou, China). All procedures were performed according to the manufacturer’s instructions. For EV-positive samples, partial VP1 gene was further amplified based on semi-nested PCR using primer pairs of 222/224 and AN88/AN89 [15]. The products were sequenced for further genotype identification as our described previously [14,16]. Patients with positive for RT-PCR but negative for semi-nested PCR were resampled to ensure successful typing of EV. Throughout the process, positive and negative controls were included to ensure the reliability of the results.

### Phylogenetic analysis

The partial VP1 genes were aligned using the ClustalW package in MEGA (version X) [17]. Bayesian evolutionary analysis sampling trees (BEAST) were then constructed based on the aligned VP1 using BEAST software (version 1.7). The tree prior used was exponential growth, and a strict clock model was applied [18]. The best-fit model, determined by jModelTest (version 2.1), was the HKY model according to the Akaike information criterion. Each Bayesian MCMC analysis was run for 30 million generations to ensure parameter convergence. The sampling frequency was set to 1 × 10^3^ generations. The output from BEAST was analysed using TRACER (v1.7.1) with ESS values higher than 200. Maximum clade credibility (MCC) trees were constructed using TreeAnnotator. The resulting trees were visualized using FigTree (version 1.4.4).

### Data analysis

Categorical variables were described using numbers and percentages. Significance values among groups were calculated using the χ^2^ test facilitated by the stats package in R Project (version 3.2.5). Trends in EV prevalence and age were analysed using the Cochran-Armitage trend test, implemented with the DescTools package. Geographic maps were created to display geographic locations of all 34 participating hospitals, enrolled cases, laboratory-confirmed cases, and the EV species for geographical variables. Heat maps were used to quantify seasonal and annual patterns of EV types based on positive cases by calendar year and month. Statistically significant results were defined as two-sided P-values less than 0.05.

## Results

### Overview

**Figure 1.**
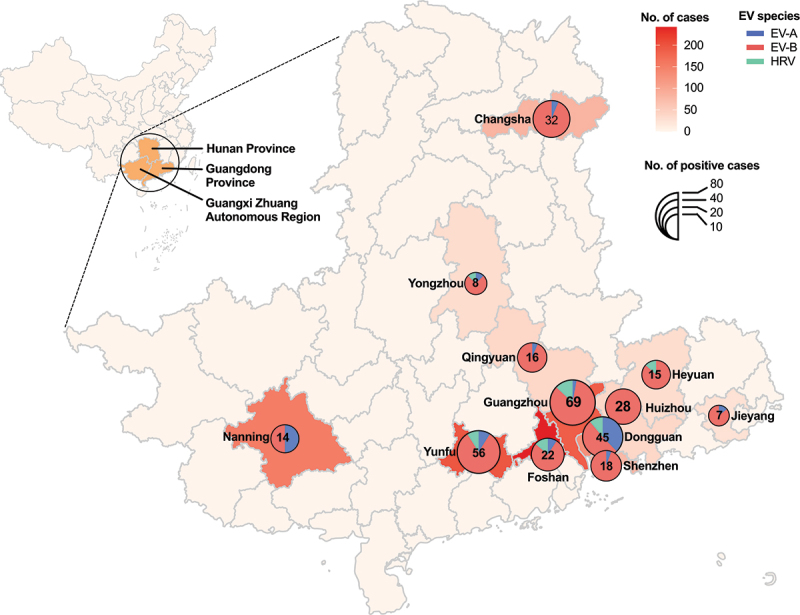
Geographic distribution of EV species in infants with suspected and confirmed EV infection across 12 cities in southern China, 2019–22. The pie charts show the proportions of EV-A (blue), EV-B (red), and HRV (green) in each city. The pie chart size reflects the number of EV species detected. EV, enterovirus; HRV, human rhinovirus.

During the 4-year study period, a total of 1221 infants with suspected EV infection from 12 cities across southern China were included in the final analysis (Figure 1). Out of these infants, 701 (57.41%) were male, 1069 (87.55%) were newborns aged 0–28 days, and 99 (8.11%) and 53 (4.34%) were infants aged 29 days–3 months and 4–12 months, respectively.

Totally, 330 (27.03%) infants were identified with EV infection. Guangzhou city had the highest number of EV infections, with 69 cases (20.91%), followed by Yunfu with 56 cases (16.97%) and Dongguan with 45 cases (13.64%). In terms of EV-positivity rate, Huizhou city had the highest rate with 75.68% (28/37), followed by Heyuan with 48.39% (15/31), Qingyuan with 43.24% (16/37), and Changsha with 40.00% (32/80). On the other hand, Foshan had the lowest EV-positivity rate of 9.09% (22/242). Detailed distribution of EV-positivity rates in different cities are shown in Table 1.

**Table 1.** Distribution of EV in infants with suspected and confirmed EV infection across 12 cities in southern China, 2019–22.

City	Suspected cases	EV-positive cases (%)	EV-A						EV-B															HRV
			CVA2	CVA4	CVA5	CVA6	CVA10	CVA16	CVA9	CVB1	CVB2	CVB3	CVB4	E3	E6	E7	E9	E11	E13	E18	E21	E25	E30	
Changsha city	80	32 (40.00)						2			2	7		2			1	8		4		1	5	
Yongzhou city	31	8 (25.81)				1					1	4									1			1
Dongguan city	128	45 (35.16)	3		1	6	5	2	1		2	9						6		2			3	5
Foshan city	242	22 (9.09)				1		1			2	2				1		6		3			3	3
Guangzhou city	187	69 (36.9)				1		1	1		5	11		1				24		8		3	5	9
Heyuan city	31	15 (48.39)										9				1		2		1				2
Huizhou city	37	28 (75.68)							2		2	6			6			7		5				
Jieyang city	24	7 (29.17)						1						1						5				
Qingyuan city	37	16 (43.24)	1									5						1		4	1		4	
Shenzhen city	77	18 (23.38)					1		1	1	1	5	2	1				4		2				
Yunfu city	196	56 (28.57)	1			1		3			3	10			1			7	1	21			3	5
Nanning city	151	14 (9.27)		1		6				1	1	1		2						1			1	
Total	1221	330 (27.03)	5	1	1	16	6	10	5	2	19	69	2	7	7	2	1	65	1	56	2	4	24	25

EV, enterovirus; CVA, coxsackievirus A; CVB, coxsackievirus B; E, echovirus; HRV, human rhinovirus.

### Type distribution

**Figure 2.**
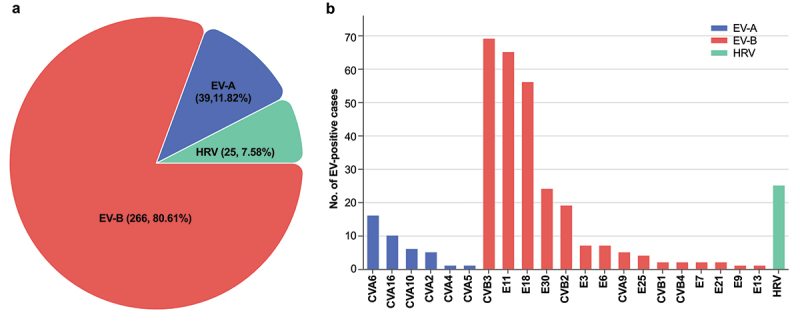
EV detection by species (a) and types (b) across 12 cities in southern China, 2019–22. EV, enterovirus; HRV, human rhinovirus; CVA, coxsackievirus A; CVB, coxsackievirus B; E, echovirus.

A total of 22 EV types were identified from the 330 EV-positive infants. Of these, 80.61% (266/330, representing 15 EV types) belonging to EV-B species, followed by EV-A (39 [11.82%], representing six types), and the remaining cases belonging to human rhinovirus (HRV) species (Figure 2a). The geographic distribution of EV species was also shown in Figure 1.

In terms of EV types, CVB3 was the most frequent type (20.90%, 69/330), followed by E11 (19.71%, 65/330), E18 (16.97%, 56/330), RV (7.58%, 25/330), E30 (7.27%, 24/330), CVB2 (5.76%, 19/330), CVA6 (4.85%, 16/330), CVA16 (3.03%, 10/330), E3 (2.12%, 7/330), E6 (2.12%, 7/330), CVA10 (1.82%, 6/330), CVA9 (1.52%, 5/330). Rare types (with fewer than five cases) including CVA4, CVA5, CVB1, CVB2, CVB4, E7, E9, E13, E21, and E25 were also identified (Figure 2b).

### The temporal pattern and seasonality

**Figure 3.**
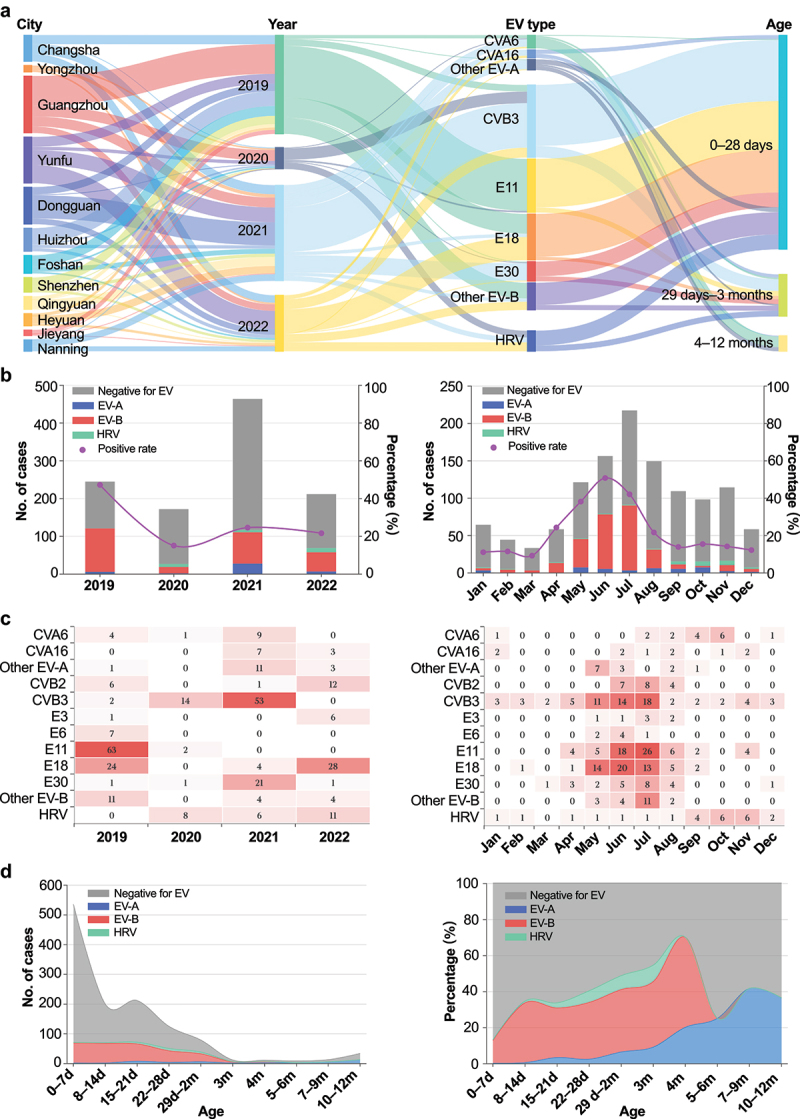
Distribution of EV by type, year, month, and age group across 12 cities in southern China, 2019–22. (a) Sankey diagram illustrating the relationships between city, year, age group, and EV type. (b) Line chart showing the trends of suspected and confirmed EV infections by year and month. (c) Heat map showing the frequencies of EV types by year and month, standardized by the annual and monthly case numbers. (d) Pie chart showing the proportions of suspected and confirmed EV infections based on different. EV, enterovirus; HRV, human rhinovirus; CVA, coxsackievirus A; CVB, coxsackievirus B; E, echovirus; d, days; m, months.

Figure 3a–c shows the temporal patterns for EV infection. The prevalence of EV decreased significantly from 47.06% (120/255) in 2019 to 14.94% (26/174) in 2020 (*p* < 0.001) due to the impact of the COVID-19 pandemic (Figure 3b). However, it then stabilized at 24.42% (116/475) in 2021 and 21.45% (68/317) in 2022 (*p* > 0.05). E11 was the most frequent EV type in 2019 (52.50%, 63/120), whereas CVB3 predominated in 2020 (70.00%,14/20) and 2021 (45.69%, 53/116), and E18 predominated in 2022 (41.18%, 28/68) (Figure 3a,c). Additionally, E18 (20.00%, 24/120) and E30 (18.10%, 21/116) were the second most frequent types in 2019 and 2021, respectively.

As shown in Figure 3b, EV infection had obvious seasonal characteristic. EV circulated mainly during warm months from May to August; June had the highest positivity rate (50.64%, 79/156), and July had the highest number of positive cases (27.58%, 91/330). However, the seasonal patterns of different species were slightly different (Figure 3c). The epidemic period of EV-B, mainly including E11, E18, and CVB3, occurred during late spring and summer with an epidemic peak in June to July. Even though the positive cases were relatively small, EV-A showed a bimodal pattern in May and October. By contrast, HRV peaked in September and November.

### Pattern of age-specific positivity rate

The relationship between EV-positive rate and age is shown in Figure 3d. EV was detected in 260 (24.32%) of 1069 newborns, 51 (51.52%) of 99 infants aged 29 days–3 months, and 19 (35.85%) of 53 older infants aged 4–12 months. EV-B declined along with the age increasing, with higher positivity rate of 88.46% (230/260) in newborns to 70.59% (36/51) infants aged 29 days–3 months to none in older infants (Z = −3.404, *p* < 0.001). In contrast, EV-A positive rates increased steadily from 4.62% (12/260) of newborn babies to 15.69% (8/51) of infants aged 29 days–3 months to 100% (19/19) of older infants (Z = 8.119, *p* < 0.01). Notably, no HRV cases were observed in infants aged 3–12 months.

### Detection of EV in different specimen types

A total of 1246 samples were collected from 1221 infants, comprising of 936 stool samples, 181 blood samples, and 129 CSF samples. The detection rate of EV in stool samples was 30.24% (283/936), which was significantly higher than the rate of 22.65% (41/181) in blood samples (*p* < 0.05), and 17.05% (22/129) in CSF samples (*p* < 0.01). However, there was no significant difference in the detection rate of EV between blood samples and CSF samples (*p* > 0.05).

### Perinatal EV transmission from mother to newborn

Table 2 presents the results of perinatal EV transmission in 16 mother-newborn pairs. Out of these pairs, EV type-specific concordance between specimens obtained from 12 newborns and their mothers, including four pairs of E11, four pairs of E6, two pairs of CVB2, one pair of CVA9, and one case of E18. Notably, five newborns (cases 4, 5, and 9 to 11) were EV positive at 1 to 2 days after birth.

**Table 2.** Analysis of vertical transmission of EV from mother to newborn.

Cases	Newborns					Mothers		
	Sex	Age	Specimen type	RT-PCR for EV	EV type	Specimen type	RT-PCR for EV	EV type
Case 1	Female	7 days	Stool	Pos.	E11	Stool	Pos.	E11
Case 2	Female	7 days	Stool	Pos.	E11			
Case 3	Female	10 days	Stool	Pos.	E6	Stool	Pos.	E6
Case 4	Female	2 days	Stool	Pos.	E6	Stool	Pos.	E6
Case 5	Male	1 day	Stool	Pos.	E6	Stool	Pos.	E6
Case 6	Female	5 days	Stool	Pos.	E6	Stool	Pos.	E6
Case 7	Female	7 days	Stool	Pos.	E11	Stool	Pos.	E11
Case 8	Female	7 days	Stool	Pos.	E11			
Case 9	Female	1 day	Stool	Pos.	CVB2	Stool	Pos.	CVB2
Case 10	Female	1 day	Stool	Pos.	CVB2	Stool	Pos.	CVB2
Case 11	Male	1 day	Stool	Pos.	E18	Stool	Pos.	E18
Case 12	Female	18 days	Stool	Pos.	CVA9	Stool	Pos.	CVA9
Case 13	Male	3 days	Stool	Neg.		Stool	Neg.	
Case 14	Male	1 day	Stool	Neg.		Stool	Neg.	
Case 15	Female	4 days	Stool+Blood	Neg.		Stool	Neg.	
Case 16	Male	1 day	Stool	Neg.		Stool	Neg.	

^a^Cases 1 and 2, and cases 7 and 8 are twins, respectively.

^b^RT-PCR, reverse transcription polymerase chain reaction; EV, enterovirus; CVA, coxsackievirus A; CVB, coxsackievirus B; E, echovirus; Pos., positive; Neg., Negative.

### Phylogenetic and evolutionary analysis

**Figure 4.**
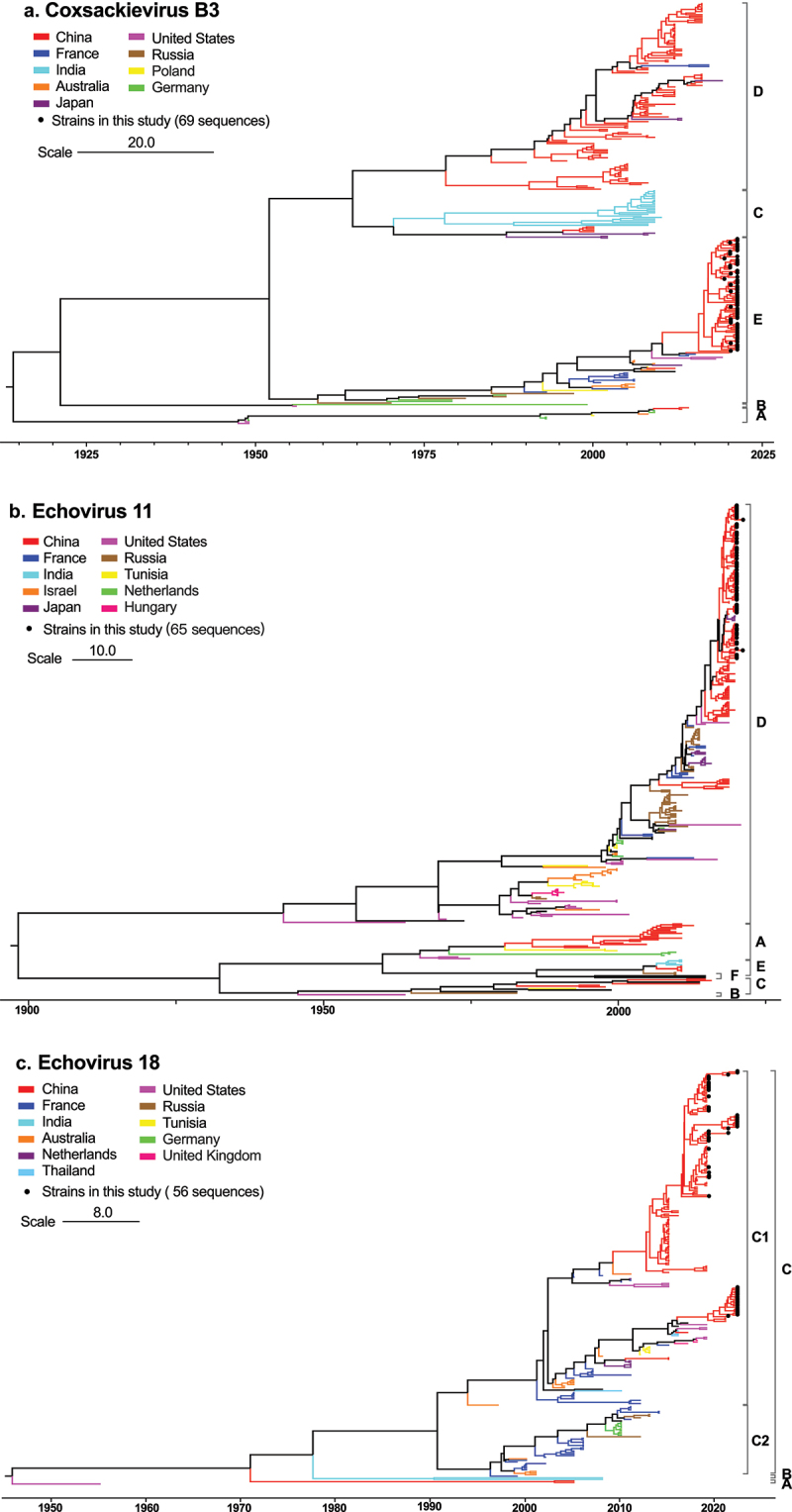
Phylogenies of CVB3 (a), E11 (b), and E18 (c) VP1 genes. Maximum clade credibility (MCC) trees of CVB3, E11 and E18 based on partial VP1 genes are shown. Black solid circles indicate the sequences from this study. The lines with different colours indicate the different countries. Scale bars indicate nucleotide substitutions per site. For clarity, the GenBank number and year of isolation of each sequence are not shown in this figure but are listed in figure S1–3 in the supplemental material. CVB, coxsackievirus B; E, echovirus.

We performed phylogenetic and evolutionary analysis of the three predominant EV types (CVB3, E11, and E18) (Supplementary Table S1) circulated in infants across southern China. Partial VP1 genes from 190 strains (69 CVB3, 65 E11, and 56 E18) in this study were analysed, along with 653 reference sequences (200 CVB3, 256 E11, and 197 E18) sampled worldwide between 1949 and 2021. The phylogenetic analysis revealed that CVB3 strains were classified into sub-type A to E, and all CVB3 strains from this study belonged to E and formed a monophyletic cluster with strains from France in 2014–2015 and from the USA in 2018–2019 (Figure 4a). For E11, a total of 312 strains were classified into six sub-types, namely A to F. The results showed that all 65 E11 strains from this cohort belonged to D and formed a monophyletic cluster with E11 strains from China between 2016 and 2018 (Figure 4b). Phylogenetic analysis also revealed that the 56 E18 strains from this study belonged to two branches of sub-type C1 (Figure 4c). Among them, 37 samples were highly similar to E18 strains from China between 2016 and 2018, while the remaining 19 strains belonged to the cosmopolitan sub-type from the USA, Germany, and Thailand between 2016 and 2019. Detailed phylogenies with GenBank accession numbers, countries of origin, and collection dates are provided in Supplementary Figure S2–4.

## Discussion

This study presents a multicenter retrospective analysis of EV data in the infant population from a large geographical area in southern China between 2019 and 2022. It reveals the epidemiological characteristics, temporal dynamics, and age-related susceptibility of different EV types in infants. The data suggest that various types of EV circulated widely in this region, with the highest detection rates observed from May to August. EV-B types were found to be the major causes of morbidity, particularly in young infants. Additionally, phylogenetic analysis was conducted to examine the evolutionary dynamics of the predominant types of CVB3, E11, and E18 in this region. Furthermore, the study includes data on maternal and neonatal infections to provide evidence of mother-to-child transmission of EV, which has been sparsely reported in the literature.

The burden of infants’ EV infection in China is still not well understood. Previous reports have suggested that EV is the main pathogen responsible for neonatal severe disorders and nosocomial outbreaks [3,14]. In this study, we found that 27.03% of infants enrolled in southern China had EV infections during the study period, with a majority of them being newborns. Similar trends have been observed in other regions, such as the United States [6] and Europe [9,19,20], where neonatal infections accounted for a significant proportion, ranging from 11% to 30%, of reported EV detections. These infections also pose a higher risk of death compared to older children [6]. Additionally, early epidemiological investigations have shown that a substantial percentage (46% − 63%) of neonates admitted due to non-bacterial fever are infected with EV [21,22]. The susceptibility of neonates to EV is primarily due to their immature immune function, lack of serotype-specific maternal antibodies, and improved diagnostic methods [23,24]. Therefore, it is recommended to routinely conduct EV-RNA detection in febrile neonates, especially during the EV season.

Using optimal specimen types for early detection can help reduce antibiotic use for infants with febrile illness. However, there is limited evidence to guide the choice of specimen type for clinical EV diagnostics. A French study found that blood samples had higher sensitivity in detecting EV in young infants with fever or sepsis-like disease compared to CSF [25]. The study suggested using blood EV testing as an early biomarker for diagnosis and management. However, it did not assess the viral diagnostic performance of stool samples. In our study, we revealed that stool samples had the highest detection rate of EV, consistent with previous study indicating that stool samples have the highest diagnostic yield for EV infection in various paediatric specimens [26]. Therefore, using stool EV-PCR testing as a diagnostic adjunct can rapidly identify young infants admitted with suspected EV, offering the added advantages of convenient collection and noninvasiveness.

Our study revealed that EV-B types were the most prevalent (80.61%) in infants, which emphasizes the significant impact of EV-B on this vulnerable age group. Among the types identified, the top three were CVB3, E11, and E18. However, it is important to note that the circulating EV types in infants can vary over time and across regions. Khetsuriani N and colleagues [6] found that that the most frequently identified types in early infancy in the United States over the past few decades were E11, CVB2, CVB5, E6, E9, and CVB4. In Europe, routine EV surveillance revealed that EV-B (i.e. CVB5, E5, E9, E11, E18, and E30) affected infants more commonly, with the dominant types varying by regions and year [9,19,20,25]. Similar results were also reported in in Iran [13] and Korea [12]. Timely and robust type-based EV surveillance has the potential to aid in the development of appropriately-targeted prevention strategies for this disease.

CVB3 is a continuously evolving virus that spreads widely. Prior to 2016, sub-type D played a major role (Figure 3a, supplement S1), supporting the previous conclusion that two lineages from D served as an important “reservoir” for the transmission of CVB3 in China [27]. However, our study found all CVB3 strains circulated between 2019 to 2022 belonged to E, revealed a sudden shift of sub-type. Interestingly, E strains distributed worldwide except in China before 2019. These findings suggest that the outbreak of CVB3 in China may have been a result of imported viruses and subsequent local transmission.

RNA viral recombination is a major driving force in the evolution and genetic architecture shaping of EV. Our previous study provided evidence suggesting that large fragment recombination could potentially contribute to the increased infectivity of E11 [14]. Similarly, Qian Yang and colleagues [27] observed a similar phenomenon in CVB3 outbreaks. However, in this study, we were unable to assess the relationship between recombination and epidemics due to the lack of complete genome data for EV strains.

The results of the present study support the notion that EV-B types are the predominant cause of EV infection in infants, particularly newborns. This finding aligns with our previous study [14] and other studies in Europe [9,25], United States [6], Korea [12] and Iran [13]. Several seroepidemiological studies have suggested that young infants have lower susceptibility and antibody levels for EV-A types due to maternal antibodies, but these levels decline quickly [28–31]. On the other hand, young infants have lower seropositivity and antibody levels for some EV-B types (such as CVB5, E6, and E30) compared to other age groups [24]. This difference may explain why young infants are more susceptible to EV-B infection.

EV can be present throughout the year, but they tend to peak during the summer or autumn months. Different species of EV exhibit different seasonal distribution patterns [32]. Our study found that EV-B displayed a more distinct peak during the summer compared to EV-A. This finding aligns with previous studies conducted in Korea [12], Spain [20], and the United States [32], where E and CV-B were primarily observed during the summer. However, Xing et al. [4] observed that climatic factors appeared to influence the seasonal pattern of EV-A, and reported that EV-A had a peak in June in north China, but experienced semi-annual outbreaks in southern China. This semi-annual pattern has also been noted in previous studies [32–34]. Although EV-A cases were infrequent in our study, they primarily occurred between May and October and exhibited a slight bimodal trend. The seasonal differences among EV species are not yet clear and require further investigation.

EV can be transmitted to the foetus through placental blood circulation, foetal inhalation or swallowing of virus particles in amniotic fluid, causing intrauterine infection. However, perinatal transmission of EV is seldom reported worldwide, and mostly as individual cases [35,36]. In this study, we observed the same EV types in the faeces of both mothers and their EV-positive newborns. Specifically, four newborns showed the presence of EV types on the first day after birth, and one newborn showed it on the second day after birth. These findings confirm the possibility of vertical transmission of EV. Few available early studies have indicated that intrapartum infection may the primary route for neonatal perinatal EV infection [37,38]. This occurs when the newborn is exposed to infectious genital secretions, amniotic fluid, blood and faeces of the mother during delivery. Additionally, postnatal exposure to oral secretions after delivery and transmission from household contacts may also contribute to neonatal EV infection [39].

Our study has several limitations that should be acknowledged. Firstly, our surveillance data primarily relied on laboratory test requests, which did not provide detailed clinical features of the infants. This hindered our ability to assess the relationship between patient disease risk or progression and EV infection types. However, it is important to note that this limitation is common to surveillance studies with similar designs [7,12]. Secondly, only fewer cities in Hunan Province and Guangxi Zhuang Autonomous Region were monitored, compared to Guangdong Province, resulting in a smaller number of enrolled infants in these regions. This limitation may have influenced our understanding of the epidemiology of EV in this specific region. Lastly, the viral phylogenetic analysis of viral phylogenetic was limited to the partial VP1 gene, which may not encompass the genome information necessary for future vaccine development or other interventions. Previous study has shown that modular intertypic recombination plays a crucial role in the evolution of EV viruses and the emergence of new strains [40]. Therefore, further studies should focus on incorporating whole-genome sequencing to gain a more comprehensive understanding of EV.

## Conclusions

In conclusion, we conducted a comprehensive and detailed investigation of the epidemiological characteristics of EV in the infant population in southern China. Our study clearly demonstrated the wide circulation of different types of EV in the region and the vulnerability of neonatal population to EV-B. Since the predominant types of EV varied from year to year, it is crucial to monitor the emergence and spread of EV in infants on a large scale and over a long period. This monitoring will help in assessing the future transmissibility of epidemics, predicting potential trends of epidemic spreading, and developing effective prevention and control measures.

## Supplementary Material

Supplemental Material

## Data Availability

All the data supporting the findings of this study are provided in the article and its Supplementary file. Additional data are available upon request from the corresponding author.
