# Supplementary material for: Emergence and circulation of enterovirus B species in infants in southern China: A multicenter retrospective analysis
Source: Virulence. 2024 Mar 31;15(1):2329569. doi: 10.1080/21505594.2024.2329569 (PMC10984118; doi:10.1080/21505594.2024.2329569)
Supplement: Supplemental Material [file KVIR_A_2329569_SM2023.zip › Supplemental_figures.docx]

**Supplemental figures**

**Figure S1.** Locations of the 34 hospitals in 12 cities across southern China from 2019 to 2022. Each point represents the location of a hospital. The background color indicates the number of enrolled cases with suspected EV infection in each city.

**Figure S2.** Maximum clade credibility (MCC) tree of CVB3 based on the partial VP1 gene. The partial VP1 genes of CVB3 (n=69) obtained from infants in this study are marked by black solid circles. Additionally, 200 reference sequences from various locations were retrieved from GenBank ([www.ncbi.nlm.nih.gov](http://www.ncbi.nlm.nih.gov)). The reference sequences are presented as GenBank accession number/country/year of isolation. The lines with different colors represent different countries. Scale bars signify 20.0 nucleotide substitutions per site.

**Figure S3.** Maximum clade credibility (MCC) tree of E11 based on the partial VP1 gene. The partial VP1 genes of E11 (n=65) obtained from infants in this study are marked by black solid circles. Additionally, 256 reference sequences from various locations were retrieved from GenBank ([www.ncbi.nlm.nih.gov](http://www.ncbi.nlm.nih.gov)). The reference sequences are presented as GenBank accession number/country/year of isolation. The lines with different colors represent different countries. Scale bars signify 20.0 nucleotide substitutions per site.

**Figure S4.** Maximum clade credibility (MCC) tree of E18 based on the partial VP1 gene. The partial VP1 genes of E18 (n=56) obtained from infants in this study are marked by black solid circles. Additionally, 197 reference sequences from various locations were retrieved from GenBank ([www.ncbi.nlm.nih.gov](http://www.ncbi.nlm.nih.gov)). The reference sequences are presented as GenBank accession number/country/year of isolation. The lines with different colors represent different countries. Scale bars signify 8.0 nucleotide substitutions per site.

Figure S1

Figure S2

Figure S3

Figure S4
