# Supplementary material for: Emergence and circulation of enterovirus B species in infants in southern China: A multicenter retrospective analysis
Source: Virulence. 2024 Mar 31;15(1):2329569. doi: 10.1080/21505594.2024.2329569 (PMC10984118; doi:10.1080/21505594.2024.2329569)
Supplement: Supplemental Material [file KVIR_A_2329569_SM2023.zip › Supplemental_Table.docx]

Table S1. Detailed information for the coxsackievirus B3, echovirus 11, and echovirus 18 strains obtained across 12 cities in southern China, 2019–22

| Strain | Enterovirus type | GenBank accession number | Sample type | City | Collection date |
| --- | --- | --- | --- | --- | --- |
| 001/GZ/GD/CHN/2019 | coxsackievirus B3 | PP097926 | stool | China: Guangzhou | 2019-04 |
| 002/GZ/GD/CHN/2019 | coxsackievirus B3 | PP097927 | stool | China: Guangzhou | 2019-04 |
| 003/SZ/GD/CHN/2020 | coxsackievirus B3 | PP097928 | stool | China: Shenzhen | 2020-01 |
| 004/GZ/GD/CHN/2020 | coxsackievirus B3 | PP097929 | stool | China: Guangzhou | 2020-02 |
| 005/FS/GD/CHN/2020 | coxsackievirus B3 | PP097930 | stool | China: Foshan | 2020-03 |
| 006/FS/GD/CHN/2020 | coxsackievirus B3 | PP097931 | stool | China: Foshan | 2020-03 |
| 007/GZ/GD/CHN/2020 | coxsackievirus B3 | PP097932 | cerebrospinal fluid | China: Guangzhou | 2020-09 |
| 008/GZ/GD/CHN/2020 | coxsackievirus B3 | PP097933 | stool | China: Guangzhou | 2020-07 |
| 009/GZ/GD/CHN/2020 | coxsackievirus B3 | PP097934 | stool | China: Guangzhou | 2020-10 |
| 010/HZ/GD/CHN/2020 | coxsackievirus B3 | PP097935 | stool | China: Huizhou | 2020-11 |
| 011/YF/GD/CHN/2020 | coxsackievirus B3 | PP097936 | stool | China: Yunfu | 2020-11 |
| 012/GZ/GD/CHN/2020 | coxsackievirus B3 | PP097937 | stool | China: Guangzhou | 2020-11 |
| 013/GZ/GD/CHN/2020 | coxsackievirus B3 | PP097938 | stool | China: Guangzhou | 2020-11 |
| 014/YZ/HuN/CHN/2020 | coxsackievirus B3 | PP097939 | cerebrospinal fluid | China: Yongzhou | 2020-12 |
| 015/HZ/GD/CHN/2020 | coxsackievirus B3 | PP097940 | stool | China: Huizhou | 2020-12 |
| 016/YF/GD/CHN/2020 | coxsackievirus B3 | PP097941 | stool | China: Yunfu | 2020-12 |
| 017/DG/GD/CHN/2021 | coxsackievirus B3 | PP097942 | stool | China: Dongguan | 2021-01 |
| 018/YF/GD/CHN/2021 | coxsackievirus B3 | PP097943 | blood | China: Yunfu | 2021-01 |
| 019/CS/HuN/CHN/2021 | coxsackievirus B3 | PP097944 | stool | China: Changsha | 2021-02 |
| 020/CS/HuN/CHN/2021 | coxsackievirus B3 | PP097945 | stool | China: Changsha | 2021-02 |
| 021/HZ/GD/CHN/2021 | coxsackievirus B3 | PP097946 | stool | China: Huizhou | 2021-04 |
| 022/YZ/HuN/CHN/2021 | coxsackievirus B3 | PP097947 | stool | China: Yongzhou | 2021-04 |
| 023/HZ/GD/CHN/2021 | coxsackievirus B3 | PP097948 | stool | China: Huizhou | 2021-04 |
| 024/QY/GD/CHN/2021 | coxsackievirus B3 | PP097949 | stool | China: Qingyuan | 2021-05 |
| 025/QY/GD/CHN/2021 | coxsackievirus B3 | PP097950 | stool | China: Qingyuan | 2021-05 |
| 026/GZ/GD/CHN/2021 | coxsackievirus B3 | PP097951 | stool | China: Guangzhou | 2021-05 |
| 027/QY/GD/CHN/2021 | coxsackievirus B3 | PP097952 | stool | China: Qingyuan | 2021-05 |
| 028/CS/HuN/CHN/2021 | coxsackievirus B3 | PP097953 | cerebrospinal fluid | China: Changsha | 2021-05 |
| 029/CS/HuN/CHN/2021 | coxsackievirus B3 | PP097954 | stool | China: Changsha | 2021-05 |
| 030/CS/HuN/CHN/2021 | coxsackievirus B3 | PP097955 | stool | China: Changsha | 2021-05 |
| 031/HZ/GD/CHN/2021 | coxsackievirus B3 | PP097956 | stool | China: Huizhou | 2021-05 |
| 032/GZ/GD/CHN/2021 | coxsackievirus B3 | PP097957 | stool | China: Guangzhou | 2021-05 |
| 033/HY/GD/CHN/2021 | coxsackievirus B3 | PP097958 | stool | China: Heyuan | 2021-05 |
| 034/HY/GD/CHN/2021 | coxsackievirus B3 | PP097959 | stool | China: Heyuan | 2021-05 |
| 035/DG/GD/CHN/2021 | coxsackievirus B3 | PP097960 | stool | China: Dongguan | 2021-06 |
| 036/YZ/HuN/CHN/2021 | coxsackievirus B3 | PP097961 | stool | China: Yongzhou | 2021-06 |
| 037/QY/GD/CHN/2021 | coxsackievirus B3 | PP097962 | stool | China: Qingyuan | 2021-06 |
| 038/DG/GD/CHN/2021 | coxsackievirus B3 | PP097963 | stool | China: Dongguan | 2021-06 |
| 039/HY/GD/CHN/2021 | coxsackievirus B3 | PP097964 | stool | China: Heyuan | 2021-06 |
| 040/NN/GX/CHN/2021 | coxsackievirus B3 | PP097965 | blood | China: Nanning | 2021-06 |
| 041/YF/GD/CHN/2021 | coxsackievirus B3 | PP097966 | blood | China: Yunfu | 2021-06 |
| 042/SZ/GD/CHN/2021 | coxsackievirus B3 | PP097967 | cerebrospinal fluid | China: Shenzhen | 2021-06 |
| 043/CS/HuN/CHN/2021 | coxsackievirus B3 | PP097968 | stool | China: Changsha | 2021-06 |
| 044/SZ/GD/CHN/2021 | coxsackievirus B3 | PP097969 | stool | China: Shenzhen | 2021-06 |
| 045/GZ/GD/CHN/2021 | coxsackievirus B3 | PP097970 | stool | China: Guangzhou | 2021-06 |
| 046/HY/GD/CHN/2021 | coxsackievirus B3 | PP097971 | stool | China: Heyuan | 2021-06 |
| 047/SZ/GD/CHN/2021 | coxsackievirus B3 | PP097972 | cerebrospinal fluid | China: Shenzhen | 2021-06 |
| 048/DG/GD/CHN/2021 | coxsackievirus B3 | PP097973 | stool | China: Dongguan | 2021-06 |
| 049/CS/HuN/CHN/2021 | coxsackievirus B3 | PP097974 | cerebrospinal fluid | China: Changsha | 2021-07 |
| 050/HY/GD/CHN/2021 | coxsackievirus B3 | PP097975 | stool | China: Heyuan | 2021-07 |
| 051/HY/GD/CHN/2021 | coxsackievirus B3 | PP097976 | stool | China: Heyuan | 2021-07 |
| 052/YZ/HuN/CHN/2021 | coxsackievirus B3 | PP097977 | stool | China: Yongzhou | 2021-07 |
| 053/SZ/GD/CHN/2021 | coxsackievirus B3 | PP097978 | cerebrospinal fluid | China: Shenzhen | 2021-07 |
| 054/HY/GD/CHN/2021 | coxsackievirus B3 | PP097979 | stool | China: Heyuan | 2021-07 |
| 055/HY/GD/CHN/2021 | coxsackievirus B3 | PP097980 | stool | China: Heyuan | 2021-07 |
| 056/HZ/GD/CHN/2021 | coxsackievirus B3 | PP097981 | stool | China: Huizhou | 2021-07 |
| 057/QY/GD/CHN/2021 | coxsackievirus B3 | PP097982 | blood | China: Qingyuan | 2021-07 |
| 058/DG/GD/CHN/2021 | coxsackievirus B3 | PP097983 | blood | China: Dongguan | 2021-07 |
| 059/DG/GD/CHN/2021 | coxsackievirus B3 | PP097984 | stool | China: Dongguan | 2021-07 |
| 060/YF/GD/CHN/2021 | coxsackievirus B3 | PP097985 | stool | China: Yunfu | 2021-07 |
| 061/HY/GD/CHN/2021 | coxsackievirus B3 | PP097986 | stool | China: Heyuan | 2021-07 |
| 062/YF/GD/CHN/2021 | coxsackievirus B3 | PP097987 | stool | China: Yunfu | 2021-07 |
| 063/YF/GD/CHN/2021 | coxsackievirus B3 | PP097988 | stool | China: Yunfu | 2021-07 |
| 064/YF/GD/CHN/2021 | coxsackievirus B3 | PP097989 | stool | China: Yunfu | 2021-07 |
| 065/DG/GD/CHN/2021 | coxsackievirus B3 | PP097990 | stool | China: Dongguan | 2021-07 |
| 066/DG/GD/CHN/2021 | coxsackievirus B3 | PP097991 | cerebrospinal fluid | China: Dongguan | 2021-08 |
| 067/YF/GD/CHN/2021 | coxsackievirus B3 | PP097992 | stool | China: Yunfu | 2021-08 |
| 068/YF/GD/CHN/2021 | coxsackievirus B3 | PP097993 | stool | China: Yunfu | 2021-09 |
| 069/DG/GD/CHN/2021 | coxsackievirus B3 | PP097994 | stool | China: Dongguan | 2021-10 |
| 070/GZ/GD/CHN/2019 | echovirus 11 | PP097995 | stool | China: Guangzhou | 2019-04 |
| 071/GZ/GD/CHN/2019 | echovirus 11 | PP097996 | stool | China: Guangzhou | 2019-04 |
| 072/GZ/GD/CHN/2019 | echovirus 11 | PP097997 | stool | China: Guangzhou | 2019-04 |
| 073/GZ/GD/CHN/2019 | echovirus 11 | PP097998 | stool | China: Guangzhou | 2019-04 |
| 074/GZ/GD/CHN/2019 | echovirus 11 | PP097999 | stool | China: Guangzhou | 2019-05 |
| 075/GZ/GD/CHN/2019 | echovirus 11 | PP098000 | stool | China: Guangzhou | 2019-05 |
| 076/GZ/GD/CHN/2019 | echovirus 11 | PP098001 | stool | China: Guangzhou | 2019-05 |
| 077/HZ/GD/CHN/2019 | echovirus 11 | PP098002 | stool | China: Huizhou | 2019-05 |
| 078/HZ/GD/CHN/2019 | echovirus 11 | PP098003 | stool | China: Huizhou | 2019-05 |
| 079/GZ/GD/CHN/2019 | echovirus 11 | PP098004 | stool | China: Guangzhou | 2019-06 |
| 080/HZ/GD/CHN/2019 | echovirus 11 | PP098005 | stool | China: Huizhou | 2019-06 |
| 081/HZ/GD/CHN/2019 | echovirus 11 | PP098006 | stool | China: Huizhou | 2019-06 |
| 082/FS/GD/CHN/2019 | echovirus 11 | PP098007 | stool | China: Foshan | 2019-06 |
| 083/FS/GD/CHN/2019 | echovirus 11 | PP098008 | stool | China: Foshan | 2019-06 |
| 084/FS/GD/CHN/2019 | echovirus 11 | PP098009 | stool | China: Foshan | 2019-06 |
| 085/YF/GD/CHN/2019 | echovirus 11 | PP098010 | blood | China: Yunfu | 2019-06 |
| 086/YF/GD/CHN/2019 | echovirus 11 | PP098011 | blood | China: Yunfu | 2019-06 |
| 087/FS/GD/CHN/2019 | echovirus 11 | PP098012 | stool | China: Foshan | 2019-06 |
| 088/HY/GD/CHN/2019 | echovirus 11 | PP098013 | stool | China: Heyuan | 2019-06 |
| 089/HY/GD/CHN/2019 | echovirus 11 | PP098014 | stool | China: Heyuan | 2019-06 |
| 090/FS/GD/CHN/2019 | echovirus 11 | PP098015 | stool | China: Foshan | 2019-06 |
| 091/GZ/GD/CHN/2019 | echovirus 11 | PP098016 | stool | China: Guangzhou | 2019-06 |
| 092/GZ/GD/CHN/2019 | echovirus 11 | PP098017 | stool | China: Guangzhou | 2019-06 |
| 093/YF/GD/CHN/2019 | echovirus 11 | PP098018 | blood | China: Yunfu | 2019-06 |
| 094/GZ/GD/CHN/2019 | echovirus 11 | PP098019 | stool | China: Guangzhou | 2019-06 |
| 095/GZ/GD/CHN/2019 | echovirus 11 | PP098020 | stool | China: Guangzhou | 2019-06 |
| 096/GZ/GD/CHN/2019 | echovirus 11 | PP098021 | stool | China: Guangzhou | 2019-06 |
| 097/YF/GD/CHN/2019 | echovirus 11 | PP098022 | blood | China: Yunfu | 2019-07 |
| 098/GZ/GD/CHN/2019 | echovirus 11 | PP098023 | stool | China: Guangzhou | 2019-07 |
| 099/SZ/GD/CHN/2019 | echovirus 11 | PP098024 | stool | China: Shenzhen | 2019-07 |
| 100/GZ/GD/CHN/2019 | echovirus 11 | PP098025 | stool | China: Guangzhou | 2019-07 |
| 101/YF/GD/CHN/2019 | echovirus 11 | PP098026 | stool | China: Yunfu | 2019-07 |
| 102/GZ/GD/CHN/2019 | echovirus 11 | PP098027 | stool | China: Guangzhou | 2019-07 |
| 103/YF/GD/CHN/2019 | echovirus 11 | PP098028 | blood | China: Yunfu | 2019-07 |
| 104/GZ/GD/CHN/2019 | echovirus 11 | PP098029 | stool | China: Guangzhou | 2019-07 |
| 105/SZ/GD/CHN/2019 | echovirus 11 | PP098030 | blood | China: Shenzhen | 2019-07 |
| 106/HZ/GD/CHN/2019 | echovirus 11 | PP098031 | stool | China: Huizhou | 2019-07 |
| 107/GZ/GD/CHN/2019 | echovirus 11 | PP098032 | stool | China: Guangzhou | 2019-07 |
| 108/CS/HuN/CHN/2019 | echovirus 11 | PP098033 | stool | China: Changsha | 2019-07 |
| 109/CS/HuN/CHN/2019 | echovirus 11 | PP098034 | stool | China: Changsha | 2019-07 |
| 110/HZ/GD/CHN/2019 | echovirus 11 | PP098035 | stool | China: Huizhou | 2019-07 |
| 111/CS/HuN/CHN/2019 | echovirus 11 | PP098036 | stool | China: Changsha | 2019-07 |
| 112/SZ/GD/CHN/2019 | echovirus 11 | PP098037 | stool | China: Shenzhen | 2019-07 |
| 113/GZ/GD/CHN/2019 | echovirus 11 | PP098038 | stool | China: Guangzhou | 2019-07 |
| 114/GZ/GD/CHN/2019 | echovirus 11 | PP098039 | stool | China: Guangzhou | 2019-07 |
| 115/FS/GD/CHN/2019 | echovirus 11 | PP098040 | stool | China: Foshan | 2019-07 |
| 116/CS/HuN/CHN/2019 | echovirus 11 | PP098041 | blood | China: Changsha | 2019-07 |
| 117/CS/HuN/CHN/2019 | echovirus 11 | PP098042 | stool | China: Changsha | 2019-07 |
| 118/QY/GD/CHN/2019 | echovirus 11 | PP098043 | stool | China: Qingyuan | 2019-07 |
| 119/CS/HuN/CHN/2019 | echovirus 11 | PP098044 | blood | China: Changsha | 2019-07 |
| 120/GZ/GD/CHN/2019 | echovirus 11 | PP098045 | stool | China: Guangzhou | 2019-07 |
| 121/CS/HuN/CHN/2019 | echovirus 11 | PP098046 | stool | China: Changsha | 2019-07 |
| 122/CS/HuN/CHN/2019 | echovirus 11 | PP098047 | blood | China: Changsha | 2019-07 |
| 123/SZ/GD/CHN/2019 | echovirus 11 | PP098048 | stool | China: Shenzhen | 2019-08 |
| 124/DG/GD/CHN/2019 | echovirus 11 | PP098049 | stool | China: Dongguan | 2019-08 |
| 125/DG/GD/CHN/2019 | echovirus 11 | PP098050 | stool | China: Dongguan | 2019-08 |
| 126/YF/GD/CHN/2019 | echovirus 11 | PP098051 | stool | China: Yunfu | 2019-08 |
| 127/GZ/GD/CHN/2019 | echovirus 11 | PP098052 | stool | China: Guangzhou | 2019-08 |
| 128/DG/GD/CHN/2019 | echovirus 11 | PP098053 | stool | China: Dongguan | 2019-08 |
| 129/DG/GD/CHN/2019 | echovirus 11 | PP098054 | stool | China: Dongguan | 2019-09 |
| 130/HZ/GD/CHN/2019 | echovirus 11 | PP098055 | stool | China: Huizhou | 2019-09 |
| 131/DG/GD/CHN/2019 | echovirus 11 | PP098056 | stool | China: Dongguan | 2019-11 |
| 132/DG/GD/CHN/2019 | echovirus 11 | PP098057 | stool | China: Dongguan | 2019-11 |
| 133/GZ/GD/CHN/2020 | echovirus 11 | PP098058 | stool | China: Guangzhou | 2020-11 |
| 134/GZ/GD/CHN/2020 | echovirus 11 | PP098059 | blood | China: Guangzhou | 2020-11 |
| 135/JY/GD/CHN/2019 | echovirus 18 | PP098060 | stool | China: Jieyang | 2019-05 |
| 136/JY/GD/CHN/2019 | echovirus 18 | PP098061 | stool | China: Jieyang | 2019-05 |
| 137/GZ/GD/CHN/2019 | echovirus 18 | PP098062 | stool | China: Guangzhou | 2019-05 |
| 138/JY/GD/CHN/2019 | echovirus 18 | PP098063 | stool | China: Jieyang | 2019-05 |
| 139/JY/GD/CHN/2019 | echovirus 18 | PP098064 | stool | China: Jieyang | 2019-05 |
| 140/JY/GD/CHN/2019 | echovirus 18 | PP098065 | stool | China: Jieyang | 2019-05 |
| 141/GZ/GD/CHN/2019 | echovirus 18 | PP098066 | stool | China: Guangzhou | 2019-05 |
| 142/GZ/GD/CHN/2019 | echovirus 18 | PP098067 | stool | China: Guangzhou | 2019-06 |
| 143/GZ/GD/CHN/2019 | echovirus 18 | PP098068 | stool | China: Guangzhou | 2019-06 |
| 144/GZ/GD/CHN/2019 | echovirus 18 | PP098069 | stool | China: Guangzhou | 2019-06 |
| 145/YF/GD/CHN/2019 | echovirus 18 | PP098070 | stool | China: Yunfu | 2019-06 |
| 146/GZ/GD/CHN/2019 | echovirus 18 | PP098071 | stool | China: Guangzhou | 2019-06 |
| 147/HZ/GD/CHN/2019 | echovirus 18 | PP098072 | stool | China: Huizhou | 2019-07 |
| 148/CS/HuN/CHN/2019 | echovirus 18 | PP098073 | stool | China: Changsha | 2019-07 |
| 149/HZ/GD/CHN/2019 | echovirus 18 | PP098074 | stool | China: Huizhou | 2019-07 |
| 150/DG/GD/CHN/2019 | echovirus 18 | PP098075 | stool | China: Dongguan | 2019-07 |
| 151/SZ/GD/CHN/2019 | echovirus 18 | PP098076 | stool | China: Shenzhen | 2019-07 |
| 152/QY/GD/CHN/2019 | echovirus 18 | PP098077 | stool | China: Qingyuan | 2019-07 |
| 153/QY/GD/CHN/2019 | echovirus 18 | PP098078 | stool | China: Qingyuan | 2019-08 |
| 154/QY/GD/CHN/2019 | echovirus 18 | PP098079 | stool | China: Qingyuan | 2019-08 |
| 155/FS/GD/CHN/2019 | echovirus 18 | PP098080 | stool | China: Foshan | 2019-08 |
| 156/FS/GD/CHN/2019 | echovirus 18 | PP098081 | stool | China: Foshan | 2019-08 |
| 157/SZ/GD/CHN/2019 | echovirus 18 | PP098082 | stool | China: Shenzhen | 2019-08 |
| 158/YF/GD/CHN/2019 | echovirus 18 | PP098083 | stool | China: Yunfu | 2019-09 |
| 159/DG/GD/CHN/2021 | echovirus 18 | PP098084 | stool | China: Dongguan | 2021-05 |
| 160/YF/GD/CHN/2021 | echovirus 18 | PP098085 | blood | China: Yunfu | 2021-06 |
| 161/FS/GD/CHN/2021 | echovirus 18 | PP098086 | blood | China: Foshan | 2021-07 |
| 162/CS/HuN/CHN/2021 | echovirus 18 | PP098087 | stool | China: Changsha | 2021-07 |
| 163/YF/GD/CHN/2022 | echovirus 18 | PP098088 | stool | China: Yunfu | 2022-02 |
| 164/YF/GD/CHN/2022 | echovirus 18 | PP098089 | blood | China: Yunfu | 2022-04 |
| 165/YF/GD/CHN/2022 | echovirus 18 | PP098090 | blood | China: Yunfu | 2022-05 |
| 166/YF/GD/CHN/2022 | echovirus 18 | PP098091 | blood | China: Yunfu | 2022-05 |
| 167/HZ/GD/CHN/2022 | echovirus 18 | PP098092 | stool | China: Huizhou | 2022-05 |
| 168/HZ/GD/CHN/2022 | echovirus 18 | PP098093 | stool | China: Huizhou | 2022-05 |
| 169/YF/GD/CHN/2022 | echovirus 18 | PP098094 | blood | China: Yunfu | 2022-05 |
| 170/YF/GD/CHN/2022 | echovirus 18 | PP098095 | stool | China: Yunfu | 2022-05 |
| 171/CS/HuN/CHN/2022 | echovirus 18 | PP098096 | stool | China: Changsha | 2022-06 |
| 172/GZ/GD/CHN/2022 | echovirus 18 | PP098097 | stool | China: Guangzhou | 2022-06 |
| 173/YF/GD/CHN/2022 | echovirus 18 | PP098098 | blood | China: Yunfu | 2022-06 |
| 174/YF/GD/CHN/2022 | echovirus 18 | PP098099 | blood | China: Yunfu | 2022-06 |
| 175/QY/GD/CHN/2022 | echovirus 18 | PP098100 | stool | China: Qingyuan | 2022-06 |
| 176/YF/GD/CHN/2022 | echovirus 18 | PP098101 | blood | China: Yunfu | 2022-06 |
| 177/YF/GD/CHN/2022 | echovirus 18 | PP098102 | blood | China: Yunfu | 2022-06 |
| 178/YF/GD/CHN/2022 | echovirus 18 | PP098103 | stool | China: Yunfu | 2022-06 |
| 179/CS/HuN/CHN/2022 | echovirus 18 | PP098104 | stool | China: Changsha | 2022-06 |
| 180/GZ/GD/CHN/2022 | echovirus 18 | PP098105 | stool | China: Guangzhou | 2022-06 |
| 181/YF/GD/CHN/2022 | echovirus 18 | PP098106 | stool | China: Yunfu | 2022-06 |
| 182/YF/GD/CHN/2022 | echovirus 18 | PP098107 | stool | China: Yunfu | 2022-06 |
| 183/YF/GD/CHN/2022 | echovirus 18 | PP098108 | stool | China: Yunfu | 2022-06 |
| 184/YF/GD/CHN/2022 | echovirus 18 | PP098109 | stool | China: Yunfu | 2022-06 |
| 185/YF/GD/CHN/2022 | echovirus 18 | PP098110 | stool | China: Yunfu | 2022-07 |
| 186/YF/GD/CHN/2022 | echovirus 18 | PP098111 | blood | China: Yunfu | 2022-07 |
| 187/YF/GD/CHN/2022 | echovirus 18 | PP098112 | blood | China: Yunfu | 2022-07 |
| 188/NN/GX/CHN/2022 | echovirus 18 | PP098113 | blood | China: Nanning | 2022-07 |
| 189/HY/GD/CHN/2022 | echovirus 18 | PP098114 | stool | China: Heyuan | 2022-07 |
| 190/HZ/GD/CHN/2022 | echovirus 18 | PP098115 | stool | China: Huizhou | 2022-09 |
